# Commercial Biomaterial-Based Products for Tendon Surgical Augmentation: A Scoping Review on Currently Available Medical Devices

**DOI:** 10.3390/jfb16040130

**Published:** 2025-04-03

**Authors:** Marta Pluchino, Leonardo Vivarelli, Gianluca Giavaresi, Dante Dallari, Marco Govoni

**Affiliations:** 1 Reconstructive Orthopaedic Surgery and Innovative Techniques—Musculoskeletal Tissue Bank, IRCCS Istituto Ortopedico Rizzoli, 40136 Bologna, Italy; marta.pluchino@ior.it (M.P.); leonardo.vivarelli@ior.it (L.V.); dante.dallari@ior.it (D.D.); 2 Surgical Science and Technologies, IRCCS Istituto Ortopedico Rizzoli, 40136 Bologna, Italy; gianluca.giavaresi@ior.it

**Keywords:** tendon repair, tendon augmentation, medical devices, natural biomaterials, synthetic biomaterials, hybrid biomaterials

## Abstract

Tendon defect is one of the common clinical diseases related to the growing population mean age and the number of athletes. Due to an increasing demand for tendon repair surgical interventions, several tendon augmentation products, capable of guaranteeing the necessary biological and visco-elasticity properties and mechanical support, have been developed. In this regard, commercially available products may be grouped into three main categories: (i) natural, (ii) synthetic, and (iii) hybrid biomaterial-based products. Firstly, to better define the research area of this work, common search engines were employed to acquire information from reports or website portfolios of important competitors in the global tendon repair market. Secondly, public registries and bibliographic databases were also employed to analyse data from registered clinical trials and published clinical studies performed to evaluate the safety and efficacy of each product. Ten new products have been launched on the market in the last fifteen years: advantages, disadvantages, and future perspectives regarding their use for tendon augmentation treatment are discussed. Although hybrid biomaterial-based products may be considered as more oriented to the new frontiers of tendon augmentation technology, future improvements, especially focused on both mechanical properties and biocompatibility, are needed. However, scientific innovations must navigate convoluted clinical regulatory paths, which, due to high costs for investors, long development timelines, and funding shortages, hinder the translation of many scientific discoveries into routine clinical practice.

## 1. Introduction

The tendon repair market size, which is estimated to account for USD 4.7 billion by 2032, as reported by Global Market Insights [1], is driven by three main factors: (i) the rise of the geriatric population, (ii) the advancements in surgical techniques, and (iii) the growing number of tendon-related injuries, especially among manual labourers, the sporting community, and the elderly [2].

According to DelveInsight’s analysis, the United States accounted for about 12 million incident cases of tendinopathy in 2022 [3], while Bergamin et al., in a recent systematic review and meta-analysis, reported a tendon rupture incidence worldwide ranging from 80 to 90 cases per 100,000 inhabitants [4]. As reported by Millar et al. [5], in the upper extremities of the body, tendinopathy is mostly observed in the rotator cuff tendons and in the common flexors and extensors of the elbow, with an incidence in the general population of 5.5% and 0.6–0.7%, respectively. Moving to the lower anatomy, most of the tendinopathies reported involve the gluteal tendons of the hip (4.2%), the knee with the patellar tendon (1.6%), and the heel and ankle region, with the Achilles tendon and the peroneal or posterior tibial tendons (2.4%). Tendinopathy mostly affects people between 18 and 65 years, with a prevalence increasing with age and in the female population. Therefore, tendinopathy represents a broad, serious clinical issue, and an economically expensive burden on healthcare systems globally. Traumatic or overuse injuries, together with inflammatory and degenerative structural changes of tendon tissue, generate acute or chronic tendon disorders, which lead to discomfort, tenderness, stiffness, severe pain, and immobility, up to the loss of daily or sporting activities.

Tendinopathy describes a complex pathology of the tendon, characterised by changes in the microstructure, cellularity, and extracellular matrix (ECM) composition (i.e., collagen type I, proteoglycans and glycosaminoglycans), which lead to pain, reduced function and low exercise tolerance. Anatomically, in injured tendons, collagen fibres appear fragmented and disorganised, causing abnormal changes in their biomechanical behaviour [5,6]. Moreover, the pathophysiological state of the tendon is highlighted by an increase of vascularity related to an upregulation of collagen type III expression and the formation of scar tissue with limited mechanical properties [7].

Currently, traditional approaches used to treat tendon disorders encompass both conservative and surgical procedures [8].

Conservative approaches, such as oral/topic administration of non-steroidal anti-inflammatory drugs (NSAIDs), corticosteroid injections, ultrasound, and physical exercises, are considered as the first line treatment applied to relieve pain, reduce inflammation, and promote structural remodelling and repair [9]. Since tendinopathy can be considered as a failed response by the homeostasis of the tendon that leads to a pathological issue, in their early stages they can be treated with conservative treatments. On the other hand, when the degenerative process has progressed to end-stage tendon rupture–a loss of tendon continuum, which can be spontaneous without prior symptomatology or derived from a preexisting tendinopathy that failed to heal correctly–several studies have stated that non-operative treatments are less successful [2]. Therefore, surgery, which primarily aims to restore tendon integrity, is the last option in the treatment of tendinopathy that persists after exhausting all non-invasive options. 

Generally, conventional primary surgical techniques involve the suturing of the wounds or the fixation of the tendon to the bone via an open approach or arthroscopy. The sutures hold the ends of the tendon filaments in place, facilitating the healing process. Alternatively, in cases of severe lesions, the injured tissue can be replaced by the transplant of an autogenous or allogeneic tendon [10]. However, in the presence of chronic disorders these approaches largely fail, probably due to tendon weakening, muscle atrophy, and decreased range of joint motion present after the primary repair of the injured tendon [11]. Moreover, concerning cadaver donor tissues, tissue banking programs often fail to meet the demand for tendon transplants due to the growing number of patients with musculoskeletal defects or injuries who have to undergo surgical grafting. 

In this respect, regulatory oversight plays a crucial role in the timely and widespread availability of allografts, especially in the EU, where the Tissues and Cells Directive (2004/23/EC) creates significant barriers to the widespread use of these materials, especially in terms of the rigorous documentation and traceability required for each graft [12]. On the other hand, in the US, the FDA’s regulation of allografts (Public Health Service Act and Code of Federal Regulations title 21 section 1271 [13]) allows for more flexibility in terms of their use in clinical settings, although it still mandates a careful balance between safety and accessibility.

Additional techniques, such as reinforcement with suture anchors or augmentation with biological or synthetic materials, may be used to enhance the stability and strength of the repair.

Therefore, despite the significant progress in tendon research, up to the present, the management of tendinopathies is restricted to symptomatic therapy, and available strategies to treat tendon disorders may be hardly considered as long-term solutions [2].

On the other hand, the rise of novel technologies and the combination of multidisciplinary knowledge and expertise may lead to the outlining of innovative procedures to treat tendinopathies. In this respect, the development of therapeutic strategies based on tissue engineering (TE) protocols may represent a way to boost the restorative process of injured tendons and to regain their complete functionality [14,15,16].

However, although the TE approach is a well-defined paradigm based on the combination of a scaffold with living cells and/or biologically active molecules to form a cell-construct for the promotion of tissue repair and/or regeneration, to date, no TE product has been translated into routine clinical procedures.

In 2006, Dr. Robert M. Nerem [17] described TE as overpromised and underdelivered, highlighting that although the field had been studied for over three decades, a very limited number of TE products (intended as cellular constructs cultivated in static/dynamic conditions) was is use for clinical purposes.

Likewise, in the field of tendon TE, to the best authors’ knowledge, although the current scientific literature to date is rich in encouraging in vitro/in vivo multi-step differentiation protocols for the tenogenic commitment of different sources of competent cells seeded on a plethora of available biomaterials with or without biochemical/physical stimuli, no clinical trial recorded on ClinicalTrials.gov database has been completed. As a consequence, no commercial TE product is present on the market due to the lack of regulatory approval for tendon augmentation procedures.

Therefore, in this scenario, the development of tendon augmentation products consisting of natural or synthetic biomaterials, or their combination, which can guarantee adherence, migration, and proliferation for autologous cells (e.g., tendon stem cells, tenocytes), visco-elasticity properties, and mechanical support [18], appears more reasonable in terms of cost/effectiveness ratios. However, it is worth noting that the approval process of new medical devices is a convoluted pathway, especially in the EU when the new Medical Device Regulation (MDR) 2017/745 [19] became effective in May 2021. Specifically, the stricter regulatory requirements introduced by the MDR, such as the need for more robust clinical evidence, rigorous risk assessments, and post-market surveillance, can influence the timelines and costs of product development leading to delays in product launches. Moreover, even the demonstration of equivalence, a wide used approach to speed up the process of new device market approval, in the current MDR it is delineated by more meticulous requirements compared to FDA 513(i) substantial equivalence [20,21].

As a result, manufacturers must plan for lengthy clinical trials, extensive documentation, and potentially extended engagement with Notified Bodies. This extended timeline can delay product launches, meaning that companies may face a competitive disadvantage if their devices take longer to market compared to those approved under the FDA’s 513(i) pathway. Therefore, the more burdensome requirements under the MDR can influence clinical adoption in the EU. Medical professionals may hesitate to use devices that have not undergone extensive clinical trials, especially if they are perceived as new or untested. The additional regulatory hurdles may also discourage manufacturers from pursuing approval for certain devices in the EU market, particularly if they are unable to demonstrate the required level of clinical evidence. On the other hand, the FDA’s less stringent approval process may lead to the quicker adoption of devices in the US. However, the reliance on equivalence data may sometimes limit innovation, as new devices that differ significantly from existing products may face more hurdles under the FDA system.

Although “tendon augmentation” is a term with broad definitions in the literature, the general purpose of these techniques is to allow for stronger repairs, which lead to improved functional outcomes by inducing host-tissue ingrowth and tendon regeneration, thereby decreasing failure rates and morbidity [22].

The purpose of this review was to provide an update regarding commercial biomaterial-based products used for tendon augmentation-based surgery, especially shedding light on recent innovations in this field.

To this aim, a comprehensive characterisation of each product was performed, comparing the manufacturers’ declared properties with outcomes emerging from the scientific literature and clinical trial databases, which may entail benefits, as well as potential drawbacks.

## 2. Materials and Methods

### 2.1. Data Sources and Search Strategy

This review was performed by evaluating only the commercially available medical devices for tendon augmentation strategies. The search was initially performed by employing popular search engines (i.e., Google and Bing) to acquire information from reports or website portfolios of important competitors in the global tendon repair market. Using this strategy, in the last fifteen years, ten distinct commercial products were identified on the global market. Successively, based on their features, they were divided into three main classes: natural, synthetic, and hybrid biomaterial-based scaffolds. Then, to acquire data and information from clinical studies performed to evaluate the safety and efficacy of each product released on the market, a scientific literature review was conducted following the PRISMA (Preferred Reporting Items for Systematic Reviews and Meta-Analyses) Extension for Scoping Reviews (PRISMA-ScR) [23]. The final protocol was registered with the Open Science Framework on 25 March 2025 (https://doi.org/10.17605/OSF.IO/GT2AB). To identify potentially relevant documents, we performed an extensive search using the ClinicalTrials.gov, Cochrane Central Register of Controlled Trials (CENTRAL), PubMed, and Scopus databases, in February 2024; a search update was conducted at the end of November of the same year.

The following term combinations were searched: “commercial name” AND “tendon”, OR “commercial name” and “augmentation”, OR “commercial name” AND “rotator cuff tears”, OR “commercial name” AND “Achilles tears”, OR “commercial name” AND “patellar tendon tears”, OR “commercial name” AND “biceps tendon tears”, OR “commercial name” AND “quadriceps tendon tears”. Only registered clinical trials and I–III Level of Evidence [24] clinical studies were selected and discussed. Moreover, the searches were filtered, only considering commercial products launched on the market from 2009 to the end of 2024, and their related published data and/or registered clinical trials.

### 2.2. Eligibility Criteria

This review focused on providing an update regarding more recently developed commercially available medical devices for tendon augmentation. Reviews, in vitro and in vivo/pre-clinical studies, and surgical technique descriptions were excluded. Only articles written in English were selected.

### 2.3. Study Selection and Data Extraction

**Figure 1:**
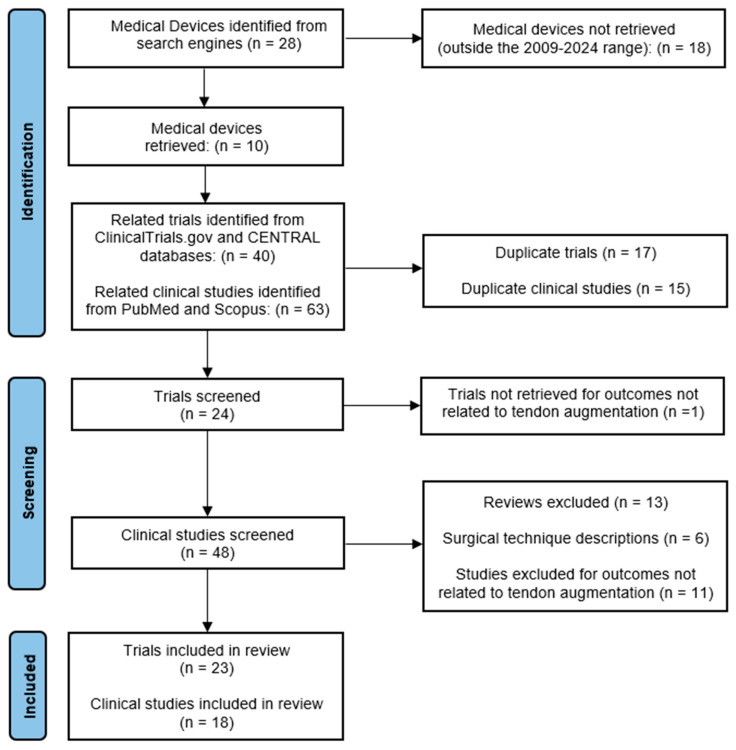
Flow chart of trials and published clinical studies included in the review.

Two reviewers–MP and MG–independently selected and evaluated the retrieved articles for eligibility. They sequentially evaluated the title and abstract and then the full text of all potentially relevant publications retrieved by the systematic search. Any disagreements were resolved by consensus. All pertinent data were extracted from the registered clinical trials and clinical studies at the level of detail reported in the Section 3. A flow chart of the selection procedure of the papers is presented in Figure 1.

## 3. Results and Discussion

**Figure 2:**
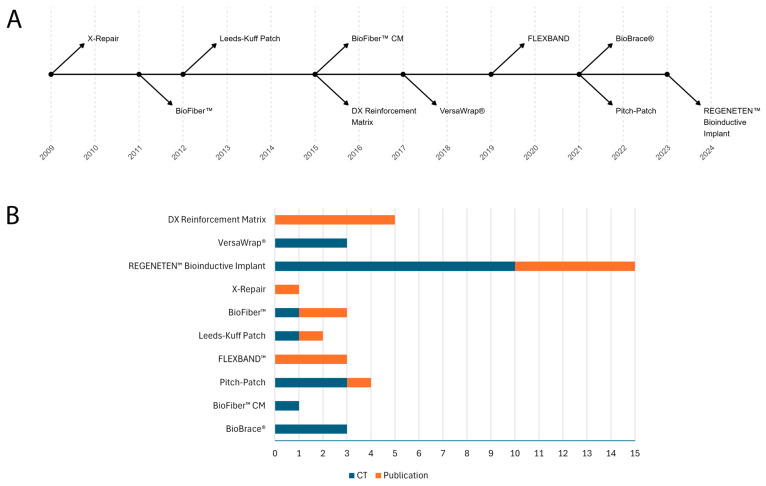
(**A**) The timeline of each medical device launched on the market between 2009 and 2024. (**B**) Number of clinical trials (in blue) and published clinical studies (in orange) involving the use of each medical device.

As listed in Table 1, Table 2 and Table 3, three natural, five synthetic, and two hybrid biomaterial-based products were launched on the market in the last fifteen years (2009–2024), as shown in Figure 2A. The original search yielded 40 clinical trials and 63 potentially relevant publications.

After the exclusion of duplicates and during the screening process (reviewing of titles and abstracts), 17 clinical trials and 45 records were excluded.

Altogether, 23 clinical trials and 18 papers—published between 2009 and 2024—met the eligibility criteria and were selected for detailed evaluation, as shown in Figure 2B.

### 3.1. Natural Biomaterial-Based Products

Scaffolds from biological sources have advantages derived from the native properties of tissue components in terms of biocompatibility, three-dimensional structure, and support for cell ingrowth. In this respect, commercial biological scaffolds used in the treatment of tendinopathies are medical devices mainly based on bovine or porcine tissues subjected to a series of cleaning treatments to remove lipids, cellular and DNA deposits, as well as cross-linking methods generally applied to obtain a scaffold that preserves the natural extracellular matrix (ECM) structure and mechanical properties [25]. Moreover, these processes are aimed at removing non-connective proteins (e.g., antigens) to prevent immune rejection in the recipient [26].

Likewise, DX Reinforcement Matrix (Arthrex Inc., Naples, FL, USA) [27] and REGENETEN™ (Smith & Nephew, Memphis, TN, USA) [28] follow the same cleaning/enzymatic treatments adopted for older medical devices since they are composed of porcine dermal ECM and bovine collagen type I, respectively.

After about ten years from the launch of DX Reinforcement Matrix on the market, only one registered clinical trial is available (i.e., NCT01586351) [29], an observational prospective clinical trial aimed at evaluating if the augmentation of rotator cuff with DX is possible using arthroscopic techniques, selectively combined with a platelet rich plasma (PRP) injection to boost the healing process, in a cohort of twenty 60-year-old patients. Although the trial has been terminated, its results have not been posted on the ClinicalTrials.gov website but they were published in 2018 by Flury et al. [30]. Specifically, the results showed that functional outcomes were similar in both groups, suggesting that patch augmentation did not bring significant benefits to patients. A retrospective study examined the use of this device in arthroscopic superior capsular reconstruction of massive and irreparable rotator cuff tears, showing an improved range of motion and a decrease in pain levels depending on the surgical treatment, not on the xenograft implant. Moreover, after a follow-up of 34 months, 25% of grafts failed [31]. Accordingly, Dr. Barber in an editorial commentary criticised the use of xenografts for rotator cuff augmentation as they fail to improve patient-reported outcomes and may even result in higher failure rates [32].

However, controversial results are reported in more recent studies where the use of DX porcine matrix showed good clinical results in patients with irreparable rotator cuff tears [33], during arthroscopic revision rotator cuff repair [34], and in a 22-year-old man affected by tarsometatarsal joint arthritis [35]. Nevertheless, it is worth noting that the lack of a control group in these studies inherently limits the ability to provide definitive evidence of treatment efficacy or safety.

REGENETEN™ Bioinductive Implant is a highly porous graft composed of collagen type I derived from highly purified bovine Achilles tendon. This product is not a novelty since it is identical to the predicate device (Collagen Tendon Sheet-DDI, K140300) developed by Rotation Medical (Plymouth, MN, USA) some years before, sharing the same physical performance characteristics, packaging, and material composition. As claimed by the manufacturer, REGENETEN™ creates an environment that permits the healing of the tissue by stimulating new tendon-like tissue formation, also permitting the augmentation of the existing tendon, and then it naturally resorbs in about six months [28].

Ten registered clinical trials have been identified, reporting the use of REGENETEN™ in rotator cuff disease [36,37,38,39,40,41,42,43,44,45]. However, only the results of the NCT04444076 [36] randomised clinical trial were published in the work of Ruiz et al. [46], who demonstrated that augmentation with REGENETEN™ of a transosseous equivalent repair in a medium-to-large posterosuperior rotator cuff tear reduced the retear rate at the 12-month follow-up by two-thirds, yielding similar improvements in clinical outcomes and without increased complication rates.

A prospective multicentre study, which enrolled 33 patients with partial-thickness rotator cuff tears treated with REGENETEN™, found that at two years of follow-up the use of this implant was safe and effective in treating tears with different grades and locations [47]. Another study compared the reoperation rates of arthroscopic repair and debridement with and without the use of this medical device in partial rotator cuff repair [48], showing that postoperative stiffness presented at a higher rate in patients that were treated with the REGENETEN™ implant than those without patch augmentation.

Interestingly, albeit it is not declared by the manufacturer, some published studies report the use of REGENETEN™ for augmentation of lower limb tendon disorders, such as chronic patellar tendon tendinopathy [49], or Achilles tendon rupture [50], showing pain reduction and complete functional recovery.

VersaWrap^®^ (Alafair Biosciences, Austin, TX, USA) [51] is a different product in terms of biomaterial sources and processing. Specifically, it is the first and, up to the present, the only plant-based bioresorbable hydrogel containing hyaluronic acid (HA) and alginate derived from the fermentation of deep ocean seaweed. VersaWrap^®^ was launched on the market in 2017 as an encasement for tendon, ligaments, skeletal muscle, and peripheral nerves.

However, to date, only three registered clinical trials have been identified [52,53,54], but they are still in the recruiting phase. Moreover, most of the published studies investigate the effects of VersaWrap^®^ are in the field of compressive neuropathies [55,56], while no clinical study on tendon repair has been published in the scientific literature.

Summary information on these medical devices is reported in Table 1.

**Table 1:** Recently developed natural biomaterial-based products commercially available on the market.

Manufacturer/ Distributor	Brand Name	Medical Device Launch (Year)	Material Type	Source	Clinical Indications	Retrieved Clinical Trials/Published Clinical Studies
Arthrex Inc., Naples, FL, USA	DX Reinforcement Matrix	2015	Dermal ECM	Porcine	Reinforcement of rotator cuff, patellar, Achilles, biceps, quadriceps and other tendons	1/5 [29]/[30,31,33,34,35]
Alafair Biosciences, Austin, TX, USA	VersaWrap^®^	2017	Hyaluronic acid and alginate	Ocean seaweed	Peripheral nerve, tendon, ligament and skeletal muscle protection	3/0 [52,53,54]/N.A.
Smith & Nephew, Memphis, TN, USA	REGENETEN™ Bioinductive Implant	2023 *	Collagen type I	Bovine	Rotator cuff disease	10/5 [36,37,38,39,40,41,42,43,44,45]/[46,47,48,49,50]

N.A. (Not Applicable): no information could be retrieved. * The predicate (i.e., Collagen Tendon Sheet-DDI) was launched on the market in 2014 by Rotation Medical.

### 3.2. Synthetic Biomaterial-Based Products

Synthetic biopolymer-based products take strength from the mechanical properties of the polymers that constitute them, providing grafts that mechanically support the tissue while healing processes take place. Polymers such as polyethylene, polyester, poly-L-lactic acid (PLLA), polyurethane–urea (PUU) and polycaprolactone (PCL), alone or in combination with other materials, represent the main synthetic materials studied as permanent reconstructions in tendon repair. Table 2 reports commercial products identified in the reviewed time-range, namely X-Repair (Synthasome, San Diego, CA, USA) [57], BioFiber™ (Wright Medical Group, Memphis, TN, USA) [58], Leads–Kuff Patch (Xiros, Leeds, UK) [59], FLEXBAND™ (Artelon, Sandy Springs, GA, USA) [60], and Pitch–Patch (Xiros, Leeds, UK) [61].

X-Repair is a PLLA biocompatible mesh with high stiffness and strength, comparable to the tendon, that is wrapped and sutured over the damaged tissue, permitting tissue integration. X-Repair is available in different sizes and it is designed to reinforce soft tissues and tendons during surgical repair procedures involving the rotator cuff, patellar, biceps, quadriceps and Achilles tendon. The PLLA of which it is composed degrades slowly, thus allowing tissue natural repair before it completely resorbs. However, there are no clinical trials discussing the use of X-Repair in tendon reinforcement procedures. Only a case series of 18 patients with large to massive rotator cuff tears was identified. This study was aimed at evaluating functional and structural outcomes of rotator cuff repair augmented with X-Repair, reporting an 83% success rate at 12 months and 78% at 42 months of follow-up after analysing imaging and clinical assessments [62]. However, as a case series, the results should be considered with caution due to the lack of a control group and the small sample size of patients enrolled in the study.

BioFiber™ is a bi-layer, synthetic absorbable reinforced woven fabric made from P4HB fibres, intended for reinforcement of soft tissues in conjunction with sutures and/or suture anchors during tendon repair surgery, including the reinforcement of rotator cuff, patellar, Achilles, biceps and quadriceps tendons. Although this medical device was launched on the market in 2011, to date, only an observational clinical trial was registered (NCT01849458) [63]. Specifically, it was a completed post-market observational study aimed at collecting and reporting data from a consecutive series of patients implanted with BioFiber™ or BioFiber™ CM (i.e., the same medical device with bovine collagen type I) as part of a post-market surveillance plan for CE Mark approval in the European Union. The results, also published on a peer-reviewed journal as an abstract, report that arthroscopic rotator cuff repair using a BioFiber™ augmented repair provides a promising option in the treatment of patients with full-thickness rotator cuff tears [64]. Recently, Burkhard et al. [65] have prospectively assessed clinical and radiologic outcomes after patch augmentation of posterosuperior rotator cuff tears using BioFiber™ patches. The results were good to excellent, with a low retear rate and good tendon integrity on 1-year postoperative magnetic resonance imaging, and the graft did not cause any complications.

Leeds–Kuff Patch and Pitch–Patch are medical devices launched on the market by Xiros (Leeds, UK) in 2012 and 2021, respectively. Both patches are made of PET, a very common thermoplastic polymer resin of the polyester family, but the most recent product features a reinforced border and incorporates reinforced-prepared holes for the sutures. For Leeds–Kuff Patch, only one clinical trial was identified, namely an interventional non-randomised study retrospectively registered on the ISRCTN database (ISRCTN79844053) [66]. Although no result is posted on this database, outcomes were recently published by Cowling et al. [67]. Specifically, they have demonstrated that any standard shoulder intervention may provide an improvement in outcome scores, but patch repair provides greater improvement in all outcomes measured by the Oxford Shoulder Score (OSS).

FlexBand^®^ is a series of products made out of degradable fibres composed of a combination of PCL and PUU knitted into a porous matrix of various configurations (bands and patches) that provides strength, elasticity and biological integration of connective tissue cells. These devices are designed to have similar strength to ankle ligaments and tendons, stretching and rebounding following foot movements, providing support for the tissue while it naturally heals. As claimed by the manufacturer, this biomaterial is quickly integrated into the damaged tissue and then degrades in 4–6 years post implantation, being gradually replaced by new connective tissue, improving patients’ recovery [60]. However, concerning the use of this medical device, no registered clinical trial was identified, while the three clinical studies recently published are limited in their conclusions regarding the efficacy of the used biomaterial due to the lack of a control group. Specifically, a retrospective study had the purpose of evaluating the safety of polycaprolactone-based polyurethane urea (PUU) devices in 105 patients who underwent foot and ankle soft tissue repair and augmentation with FlexBand^®^. The evaluation of postoperative records and radiographs at about 6 months of follow-up showed low complication rates, with only one case of graft removal [68]. In the same year, Mendes de Carvalho et al. [69] reported a case study in which a 56-year-old man with a chronic Achilles tendon rupture, treated with a flexor hallucis longus (FHL) transfer reinforced with FlexBand^®^, achieved improvements in both recovery and strength with complete integration and healing of the graft at 12 months post-surgery. The same good clinical results, with no foreign body reactions, have been observed by Solan et al. [70] in a 29-year-old man with an extensor hallucis longus (EHL) tendon laceration who was treated by adding FlexBand^®^ as an augmentation patch.

For Pitch–Patch, previously distributed by IST Innovative Surgical Technologies AG (Cham, Switzerland), three studies were registered on the ClinicalTrials.gov database. However, no result has been posted since two trials are still in the recruiting phase (NCT05906004 [71], NCT06076902 [72]), and one was withdrawn for personal reasons (NCT03511547) [73]. On the other hand, Smolen et al. [74] have investigated in a prospective cohort study the impact of Pitch–Patch augmentation for massive rotator cuff tears, showing good clinical results and leading to a substantially lower retear rate.

**Table 2:** Recently developed synthetic biomaterial-based products commercially available on the market.

**Manufacturer/** **Distributor**	**Brand Name**	**Material Type**	**Medical Device Launch (Year)**	**Clinical** **Indications**	**Retrieved Clinical** **Trials/Published Clinical Studies**
Synthasome, San Diego, CA, USA	X-Repair	Poly-L-lactic acid (PLLA)	2009	Reinforcement of soft tissues and tendons	0/1 N.A./[62]
Wright Medical Group, Memphis, TN, USA	BioFiber™	Poly-4-hydroxybutyrate fibres (P4HB)	2011	Reinforcement of rotator cuff, patellar, Achilles, biceps and quadriceps tendons	1/2 [63]/[64,65]
Xiros, Leeds, UK	Leeds–Kuff Patch	Polyethylene terephthalate (PET)	2012	Reinforcement of the rotator cuff following or during repair by suture or suture anchors	1/1 [66]/[67]
Artelon, Sandy Springs, GA, USA	FLEXBAND™	Co-polymer of polycaprolactone (PCL) and polyurethane-urea (PUU)	2019	Ankle tendon and ligament augmentation	0/3 N.A./[68,69,70]
Xiros, Leeds, UK	Pitch–Patch	Polyethylene terephthalate (PET)	2021 *	Reinforcement of the rotator cuff following or during repair by suture or suture anchors	3/1 [71,72,73]/[74]

N.A. (Not Applicable): no information could be retrieved. * Previously distributed by IST Innovative Surgical Technologies AG (Cham, Switzerland).

### 3.3. Hybrid Biomaterial-Based Products

Hybrid biopolymer-based products have been developed to benefit from the advantages of both natural and synthetics, namely biomimicking ECM and mechanical properties, respectively.

However, although many hybrid composites have been developed for tendon TE protocols, only a few products have reached the market. From 2009 to the present, only two products have been identified (Table 3): BioFiber™ CM (Wright Medical Group, Memphis, TN, USA) [75] and BioBrace^®^ (CONMED, Utica, NY, USA) [76].

In 2015, the Wright Medical Group launched on the market a bio-composite device (i.e., BioFiber™ CM), in which synthetic P4HB fibres were coated with bovine-derived collagen type I. However, the only registered clinical trial identified was the same post-market observational study performed for the device without a bovine collagen coating (NCT01849458 [63]). Moreover, no published clinical study was found in the scientific literature, making it difficult to prove the efficacy of this medical device for tendon augmentation procedures; in 2020, Stryker (Mahwah, NJ, USA) acquired Wright Medical Group, but neither BioFiber™ nor BioFiber™ CM are to date included in its portfolio.

BioBrace^®^ is a bio-composite scaffold made from biological and synthetic materials. The biological matrix composed of highly porous collagen type I is reinforced with bioresorbable PLLA microfilaments, which promotes the load sharing and healing process. Following the manufacturer’s instructions, this device can be used in knee, shoulder, hip, foot and ankle surgical procedures, both for tendons and ligaments. However, due to the scope of this review, only studies on tendon augmentation strategies were examined. In this respect, three registered clinical trials, all focusing on shoulder tendons, were identified (NCT05997381 [77], NCT05959733 [78], and NCT05487677 [79]). However, all these studies were recently registered on the ClinicalTrials.gov database and, therefore, no results have been posted yet. On the other hand, although there are several published studies in which BioBrace^®^ was used as a scaffold for tendon augmentation, they are surgical technique descriptions [80,81,82,83], and, therefore, not discussed in this review.

**Table 3:** Recently developed hybrid biomaterial-based products commercially available on the market.

Manufacturer/Distributor	Brand Name	Composition	Medical Device Launch (Year)	Clinical INDICATIONS	Retrieved Clinical trials/Published Clinical Studies
Wright Medical Group, Memphis, TN, USA	BioFiber™ CM	Bovine collagen type I and poly-4-hydroxybutyrate fibres (P4HB)	2015	Tendon and ligament repair	1/0 [63]/N.A.
CONMED, Utica, NY, USA	BioBrace^®^	Bovine collagen type I and poly-L-lactic acid (PLLA)	2021	Knee, shoulder, hip, foot and ankle tendons augmentation	3/0 [77,78,79]/N.A.

N.A. (Not Applicable): no information could be retrieved.

The following Table 4 and Table 5, respectively report the list of clinical trials and published clinical studies discussed in this review, summarising their main characteristics. Table 6 shows the key properties of each medical device, which is useful for a quick comparison among the products. Finally, Table S1, provided in the Supplementary Materials, reports all the retrieved data along with webpage links to the medical devices and their potential adverse effects.

Because most clinical studies examining medical devices for tendon and ligament repair did not perform in vivo measurements in human patients, the mechanical properties are often referred to as in vitro or in vivo testing, in particular involving cadaver or animal samples [84]. It is well-established that natural scaffolds tend to be more flexible but weaker, while synthetic scaffolds are stiffer but can be more prone to inflammatory responses, and hybrid scaffolds aim to balance these traits, offering a combination of strength, flexibility, and biocompatibility. However, despite the broad use of these scaffold types, there is a notable gap both in the available literature and the information provided by manufacturers regarding the specific mechanical properties of these medical devices. This lack of detailed data makes it difficult to assess how well a particular device will perform in different clinical scenarios. Therefore, when selecting a device for repairing a specific tendon or ligament, it is essential to consider the mechanical properties of the native tissue, including its natural force and stress characteristics, to ensure that the chosen medical device will closely match the needs of the anatomical region being repaired. The available claimed biomechanical features of each medical device are listed above in Table 6.

The main limitations of this review are related both to the clinical trials and the published clinical studies here reviewed.

Regarding registered clinical trials, low enrolment, lack of funding, and failure to achieve efficacy outcomes in interim analysis are common circumstances that can dramatically change the projected timelines affecting the interpretation of results. In this regard, it is worth noting that only 4 of 23 clinical trials here identified are concluded, while the majority of the studies are still in the recruiting phase, withdrawn, or in an unknown status. Although in orthopaedics clinical trials have to face several challenges that can complicate the process of generating reliable evidence, particularly with RCTs, practical and ethical limitations should not lead to the inclusion of less rigorous study designs. Therefore, as reported by Losina et al. [85], a cost-effectiveness analysis in orthopaedic research is critical, especially to ensure rigorous attention to the study protocol during implementation, as well as thoughtful data analysis and reporting.

**Figure 3:**
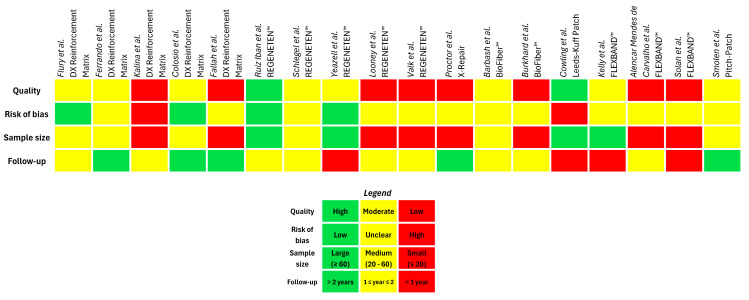
Quality assessment framework of the retrieved clinical studies related to the use of medical devices for tendon augmentation [30,31,33,34,35,46,47,48,49,50,62,64,65,67,68,69,70,74]. Quality: Green—High quality (Randomised Controlled Trial); Yellow—Moderate quality (Cohort study); Red—Low quality (Case report/Case series). Risk of bias: Green—Low risk of bias (well-conducted study with a control group); Yellow—Unclear risk of bias (insufficient information to determine the risk); Red—High risk of bias (concerns like lack of blinding and/or randomisation). Sample size: Green—Large sample size (60 or more subjects); Yellow—Medium sample size (20 to 60 subjects); Red—Small sample size (20 or fewer subjects). Follow-up: Green—>2 years (follow-up period of more than two years); Yellow—1 ≤ year ≤ 2 (follow-up period between one and two years included); Red—<1 year (follow-up period of less than one year).

Moreover, due to the lack of robust published clinical studies, this review has evaluated some case reports and retrospective studies, despite the well-known fact that the absence of a control group may hinder the establishment of a clear cause-and-effect relationship, which is crucial for drawing reliable conclusions and making evidence-based clinical decisions (Figure 3). However, their inclusion in the eligibility criteria is intended to provide the scientific community with clinical observations and outcomes that can guide clinical decision-making, particularly in terms of safety. Finally, other limitations, such as gender imbalance, the presence of a specific co-morbidity (i.e., obesity), and/or the concomitant use of drug therapy (i.e., NSAIDs or corticosteroids) could reduce the emphasis related to the outcomes.

### 3.4. Cost/Effectiveness Considerations

In tendon augmentation procedures, the use of the medical devices here reviewed potentially leads to faster recovery times, reduced re-surgeries, and improved functional outcomes, all of which ultimately lower the total cost of care by decreasing the need for follow-up interventions. While these devices typically add between $2.000 and $5.000 to the total cost of surgery, the long-term benefits often outweigh the initial expense. The enhanced healing, increased tendon strength, and reduced re-tear rates can lead to fewer revisions and a quicker return to function, making these devices a potentially cost-effective solution in complex, high-risk tendon repairs. However, despite these promising features, the cost/effectiveness ratio of such medical devices is still underexplored in the scientific literature. For instance, studies like that of Quigley et al. [86] have demonstrated that rotator cuff repairs with graft augmentation improve functional outcomes, reduce revision surgeries, and are cost-effective. Similarly, Rognoni et al. [87] reported a €4.918 saving per healed tear when using the bioinductive implant REGENETEN™ for rotator cuff repairs. Nonetheless, caution is necessary when interpreting these findings since company-specific products should be evaluated independently, as medical insurance coverage and reimbursement rates can vary greatly depending on the country. Additionally, there is no widespread consensus on cost/effectiveness data related to rotator cuff repair, much less on tendon augmentation procedures [88]. Nevertheless, further research is essential to better understand the long-term cost/effectiveness and clinical impact of these devices.

## 4. Conclusions

Considerable improvements in techniques of tendon repair have been achieved in the past decades due to a plethora of biomaterial-based products helpful for restoring the normal function and movement of the affected area [89].

As tendons are poorly perfused tissues, which heal slowly and frequently form scar tissue [90], hybrid biomaterial-based products may be considered as more oriented to new frontiers of tendon augmentation technology since they provide the advantages both of natural and synthetic biomaterials. However, in the last fifteen years, only two medical devices in this category have been launched on the market (i.e., BioBrace^®^ and BioFiber™ CM), while the most of new products show minimal innovative features, reflecting the trend that regulatory hurdles for clinically approved natural or synthetic scaffolds can be lower than those for novel biomaterials. The main reason resides in not performing biocompatibility or safety tests for devices that are substantially equivalent or identical to an existing product, while on the contrary, new materials have to undergo exhaustive in vitro/in vivo tests, which takes several additional years prior to being marketed [91].

However, further research efforts are crucial to advance this field, particularly considering that, to date, collagen type I from xenogeneic sources remains the most commonly used, yet over-relied upon, bioactive compound for coating synthetic scaffolds. In this context, REGENETEN™, a medical device that shares many characteristics with its predicate, has been utilised in several RCTs, producing published data that demonstrates its efficacy and high acceptance among orthopaedic surgeons.

On the other hand, for many of the medical devices here reviewed, accurately assessing their true safety and efficacy remains a significant challenge. The difficulty lies in the complexity of conducting comprehensive, long-term clinical studies, which are essential to confirm the performance and outcomes of tendon repair products. While many devices demonstrate initial promise in preclinical studies or smaller trials, real-world effectiveness may vary across diverse patient populations due to factors such as age, comorbidities, and the severity of tendon injuries. As a result, clinicians must exercise caution when selecting tendon repair devices, particularly newer biomaterial-based products that may lack substantial clinical evidence to support their safety and effectiveness. In clinical practice, the priority should be to choose devices with robust clinical trial data, demonstrating proven efficacy and safety over time. The incorporation of these well-established products into treatment protocols minimizes risks and ensures better patient outcomes.

Furthermore, to strengthen the body of evidence surrounding new tendon repair devices, collaboration among clinicians, researchers, and regulatory bodies is essential. In this regard, tissue engineering protocols hold significant promise for advancing tendon repair. These approaches, which integrate cell-based therapies, bioactive growth factors, and advanced biomaterial scaffolds, have the potential to create functional tendon replacements that go beyond simple mechanical repair by improving the regenerative capabilities of tendons. Such strategies could not only accelerate healing but also offer more personalized and durable solutions for patients suffering from tendon injuries. However, despite the exciting prospects of tissue engineering, these advanced therapies face a particularly stringent regulatory path. Consequently, the regulatory demands may delay the availability of these promising therapies.

Hence, to accelerate the introduction of breakthrough products into the market, it is critical to establish more efficient testing protocols that balance safety and efficacy with the need for timely approval, reducing the duration and cost of clinical trials without compromising patient safety.

## Figures and Tables

**Table 4:** Registered clinical trials testing the use of medical devices for tendon augmentation. Sources: ClinicalTrials.gov and CENTRAL.

Medical Device (Brand Name)	Clinical Trial ID	Study Focus	Intervention	Study Design	Actual Estimated Enrolment	Clinical Trial Status	Posted Results	Conflicts of Interest *
DX Reinforcement Matrix	NCT01586351 [29]	Rotator cuff tears	Patch implant and autologous conditioned plasma injection	Observational	20 20	Completed	N.A.	Likely
	NCT04444076 [36]	Supraspinatus tear	Bioinductive implant on supraspinatus tendon repair	Interventional, randomised	124 120	Active, not recruiting	N.A.	Yes
REGENETEN™	NCT04450342 [37]	Rotator cuff tears	Bioinductive implant augmentation	Interventional, prospective, multi-centre, randomised	119 300	Terminated	N.A.	Likely
	NCT04861714 [38]	Subscapularis tendon	Augmentation for subscapularis healing after total shoulder arthroplasty	Interventional, randomised	75 50	Active, not recruiting	N.A.	No
	NCT03734536 [39]	Rotator cuff tear	Surgical treatment of partial-thickness rotator cuff tears the bioinductive implant	Interventional, non-randomised	118 118	Terminated	N.A.	Yes
	NCT06252389 [40]	Achilles rupture	Achilles Tendon repair augmented with bioinductive collagen patch	Observational, retrospective case series	N.A. 9	Not yet recruiting	N.A.	No
	NCT04673344 [41]	Rotator cuff tear	Partial rotator cuff repair surgery with the addition of the bioinductive collagen patch	Interventional, randomised	N.A. 80	Unknown	N.A.	No
	NCT06269965 [42]	Rotator cuff syndrome	Arthroscopic shoulder repair, in double row, with complete coverage of the foot print and addition of the bioinductive collagen patch	Interventional, randomised	N.A. 204	Not yet recruiting	N.A.	No
	NCT04248751 [43]	Massive rotor cuff tear	Bioinductive implant augmentation	Interventional, prospective, randomised	N.A. 76	Recruiting	N.A.	Likely
	NCT05444465 [44]	High grade partial-thickness tear	Isolated Bioinductive repair with the Bioinductive Implant	Interventional, randomised	N.A. 156	Recruiting	N.A.	Yes
	NCT06215417 [45]	Rotator cuff tear	Arthroscopic rotator cuff repair augmented with graft	Interventional, randomised	N.A. 102	Not yet recruiting	N.A.	No
VersaWrap^®^	NCT05598801 [52]	Hand/fingers tendon repair	Graft applied to the affected tendon to allow post-operative gliding.	Prospective, observational	N.A. 20	Enrolling by invitation	N.A.	No
	NCT04976335 [53]	Flexor tendon	Membrane placed between distal radius plate and flexor tendons	Interventional, randomised	N.A. 100	Recruiting	N.A.	No
	NCT04322370 [54]	Zone 2 flexor tendon	Graft applied to the flexor tendon where there is no significant loss of tendon tissue	Interventional, prospective, randomised	42 52	Recruiting	N.A.	No
X-Repair	/	/	/	/	/	/	/	/
BioFiber™	NCT01849458 [63]	Full thickness rotator cuff tears	Subjects implanted with BioFiber	Post-market observational	50 50	Completed	Improvements in clinical functional outcomes	Yes
Leeds–Kuff-Patch	ISRCTN79844053 [66]	Rotator cuff tears	Patch implant	Interventional, non-randomised	68 60	Completed	Improvements in outcome scores	No
Pitch–Patch	NCT05906004 [71]	Rotator cuff tears	Patch device used for rotator cuff augmentation/ reinforcement	Observational, perspective	N.A. 32	Not yet recruiting	N.A.	Yes
	NCT06076902 [72]	Rotator cuff tears	Pitch–Patch device used for rotator cuff augmentation/ reinforcement	Interventional, prospective, randomised	N.A. 300	Recruiting	N.A.	No
	NCT03511547 [73]	Supraspinatus tendon tear	Pitch–Patch device used for rotator cuff augmentation	Interventional, randomised	0 N.A.	Withdrawn	N.A.	No
FLEXBAND™	/	/	/	/	/	/	/	/
BioBrace^®^	NCT05997381 [77]	Full thickness rotator cuff tears	Implant augmentation	Interventional, randomised	N.A. 268	Enrolling by invitation	N.A.	Yes
	NCT05959733 [78]	Rotator cuff tears	Implant augmentation	Interventional, randomised	N.A. 60	Recruiting	N.A.	Likely
	NCT05487677 [79]	Subscapularis repair	Implant augmentation	Interventional, randomised	N.A. 100	Recruiting	N.A.	No
BioFiber™ CM	NCT01849458 [63]	Full thickness rotator cuff tears	Implant augmentation	Post-market observational	50 50	Completed	Improvements in clinical functional outcomes	Yes

N.A. (Not Applicable): no information could be retrieved. * Yes: the manufacturer is the sponsor of the clinical trial; Likely: the manufacturer is a collaborator of the clinical trial; No: the manufacturer is not involved in the clinical trial.

**Table 5:** Summary of published findings of clinical studies related to the use of medical devices for tendon augmentation.

Medical Device (Brand Name)	Clinical Trial ID	Study Focus	Intervention	Control/Comparator	Study Design	Subjects (Gender, No., Age)	Results	Conflicts of Interest **	Refs.
DX Reinforcement Matrix	NCT01586351 [29]	Rotator cuff repair	ARCR + patch implant	Group without patch	Observational study	F: 28, M: 12; 60–74	No significant group differences	Potential	[30]
	/	Superior capsular reconstruction	SCR + patch implant	/	Retrospective study	F: 17, M: 39; 56–74	No significant improvement; 25% graft failure (34 months f.u.)	Yes	[31]
	/	Superior capsular reconstruction	SCR + patch implant	/	Pilot study	F: 8, M: 12; 48–73	Significant pain relief and a considerable improvement in the range of motion	N.A.	[33]
	/	Revision rotator cuff repair	ARRCR + patch implant	Group without patch	Retrospective comparative study	F:22, M: 18; 56–70	↑ CMS ↔ DASH	Yes	[34]
	/	The 5th TMT joint	Interpositional arthroplasty + patch implant	/	Case report	M: 1; 22 years	Asymptomatic patient at 3 years f.u., symmetric mobility, AOFAS of 100/100	Likely	[35]
REGENETEN™	NCT04444076 [36]	Medium-to-large posterosuperior rotator cuff tears	TOE repair + Patch implant	Group without patch	Randomised controlled trial	F: 63, M: 61; 49–62	Two-third reduction of the retear rate at 12 month f.u. Similar improvements in clinical outcomes. No increase in complication rates	Potential	[46]
	/	Intermediate- and high-grade partial-thickness rotator cuff tears	Arthroplasty with bioinductive implant	/	Prospective study	F: 14, M: 19; 33–74	↑ ASES and CMS	Yes	[47]
	/	High-grade partial-thickness rotator cuff tears	Arthroscopic debridement + bioinductive collagen patch	Group without patch	Propensity-matched trial	F: 28, M: 36; 54.4	↑ Postoperative stiffness	No	[48]
	/	Case 1: chronic patellar tendinopathy Case 2: chronic proximal hamstring tendinopathy	Cases 1 and 2: bioaugmentation in the surgical treatment of chronic tendinopathies	/	Report of two cases	Case 1: M, 22 Case 2: F, 40	Cases 1 and 2: an accelerated rate of return to patient pre-injury activity levels	No	[49]
	/	Acute Achilles tendon rupture	Bioaugmentation	/	Case report	F: 1; 16 years	Full range of motion, strength, and MRI evidence of increased tendon thickness	No	[50]
VersaWrap^®^	/	/	/	/	/	/	/	/	/
X-Repair	/	Large to massive rotator cuff tears	Arthroscopic repairs with patch reinforcement and fixation	/	Case series	Gender: N.A. No.: 18 Age: 52–89	↑ ASES	No	[62]
BioFiber™	/	Arthroscopic rotator cuff repair	Arthroscopic repairs with patch reinforcement	/	Prospective trial	F: 27, M: 23; 52–70	↑ CMS, WORC, ROM, and strength testing	N.A.	[64]
	/	Posterosuperior rotator cuff repair	Arthroscopic repairs with patch reinforcement	/	Controlled case series	F: 4, M: 12; 45–76	↑ CMS, Muscular strength	No	[65]
Leeds–Kuff-Patch	ISRCTN79844053 [66]	Large and massive rotator cuff tears	Arthroscopic repairs with patch reinforcement	Group without patch	Feasibility study	F: 42, M: 26; 56–74	↑ OSS and SPADI, ↔ CMS	Yes	[67]
Pitch–Patch	/	Massive rotator cuff tears	Arthroscopic repairs with patch reinforcement	/	Prospective cohort study	F: 16, M: 34; 57–73	↑ CMS, ↓ retear rate	No	[74]
FLEXBAND™	/	Soft tissue reconstruction in foot and ankle surgery	Tissue reconstruction with patch reinforcement	/	Retrospective study	F: 65, M: 40; 34–66	↓ VAS, low compication rate	Potential	[68]
	/	Achilles tendon reconstruction	Tissue reconstruction with patch reinforcement	/	Case report	M: 1, 56 years	↑ PROMIS GPH T-score, ↓ PROMIS Pain T-score, ↓ ALS	No	[69]
	/	Extensor hallicus longus tendon laceration	Tissue reconstruction with patch reinforcement	/	Case report	M: 1, 29 years	↑ Functional outcomes	No	[70]
BioBrace^®^	/	/	/	/	/	/	/	/	/
BioFiber™ CM	/	/	/	//	/	/	/	/	/

Abbreviations: ↑: increase; ↔: no change; ↓: decrease; ALS: Activity Limitation Score; AOFAS: American Orthopaedic Foot and Ankle Society scale; ARCR: arthroscopic rotator cuff repair; ARRCR: arthroscopic revision rotator cuff repair; ASES: American Shoulder and Elbow Surgeons scale; CMS: Constant–Murley score; DASH: disabilities of the arm, shoulder, and harm score; MRI: magnetic resonance imaging; OSS: Oxford Shoulder Score; PROMIS GPH: PROMIS Global Physical Health; ROM: range of motion; SCR: superior capsular reconstruction; SPADI: Shoulder Pain and Disability Index; TOE: transosseous equivalent; TMT: tarso-metatarsal; VAS: visual analogue scale; WORC: Western Ontario Rotator Cuff index; N.A. (Not Applicable): no information could be retrieved. ** Yes: the authors declare a conflict of interest; Potential: the authors declare a potential conflict of interest; Likely: the authors declare no conflict of interest but some authors have/had work relationships with the manufacturer; No: the authors declare they have no conflict of interest.

**Table 6:** Key features of commercial biomaterial-based products launched on the market in the last fifteen years (2009–2024).

Medical Device (Brand Name)	Category	Claimed Biomechanical Properties	Clinical Indication and Use					Shape	Dimensions
			Rotator Cuff	Patellar Tendon	Achilles Tendon	Other Tendons/Ligaments	Arthroscopy Use		
DX Reinforcement Matrix	Natural Biomaterial	Maintains strength (mean 137.5 N/cm) greater than native fascial tissue and the empty control throughout the healing process	Yes	Yes	Yes	Yes	Yes	Patch	5 × 5 cm–6 × 8 cm
REGENETEN™ Bioinductive Implant	Natural Biomaterial	N.A.	Yes				Yes	Membrane	Size of a postage stamp
VersaWrap^®^	Natural Biomaterial	Reduction of 46% in friction (peak gliding resistance analysis); the analysis of rupture strength showed that it does not interrupt the repair process				Yes	Gel only	Ultrathin membrane or gel	Membrane: 2.5 × 5 cm–5 × 5 cm Gel: 1 mL
X-Repair	Synthetic Biomaterial	Tensile modulus: 500 MPa				Yes	Yes	Patch	1.2 × 4.3 cm–2.5 × 2.5 cm–2.5 × 3 cm–2.5 × 3.5 cm–2.5 × 4.3 cm–4 × 3 cm–4 × 3.5 cm–4 × 4.3 cm
BioFiber™	Synthetic Biomaterial	Tensile strength: 2500 N	Yes	Yes	Yes	Yes	Yes	Strip or disc	Strips: 1.3 × 2.3 cm–2 × 3 cm–2.5 × 5 cm; Disc: ⌀ 0.8 cm
Leeds–Kuff Patch	Synthetic Biomaterial	Suture retention strength: 550 N	Yes				Yes	Patch	2 × 2 cm–3 × 3 cm–3.5 × 4 cm
Pitch–Patch	Synthetic Biomaterial	N.A.	Yes				Yes	Patch	3 × 2 cm–3.5 × 2.5 cm
FLEXBAND™	Synthetic Biomaterial	Provides a high suture retention strength compared to other commercially available products				Yes	No	Strip	0.3 × 8 cm–0.3 × 16 cm–0.3 × 32 cm–0.5 × 8 cm–0.5 × 16 cm–0.5 × 32 cm–0.7 × 8 cm–0.7 × 16 cm–0.7 × 32 cm
BioBrace^®^	Hybrid Biomaterial	Strength of 355 N when fully sutured along the medial and lateral edges				Yes	Yes	Patch or cord	Patch: 2.3 × 3 cm–4 × 6 cm; Cord: ⌀ 0.5 cm × 25 cm
BioFiber™ CM	Hybrid Biomaterial	Ultimate tensile strength of 172.2 N, similar to ankle ligaments				Yes	Yes	Strip	2 × 3 cm

N.A. (Not Applicable): no information could be retrieved.

## Data Availability

No new data were created or analysed in this study. Data sharing is not applicable to this article.

## Supplementary Materials

The following supporting information can be downloaded at: https://www.mdpi.com/article/10.3390/jfb16040130/s1, Table S1: Retrieved data from medical devices included in this review.
